# Complete Genome Sequence of Acidithiobacillus ferriphilus GT2, Isolated from Gold Mill Tailings

**DOI:** 10.1128/mra.01089-21

**Published:** 2022-02-03

**Authors:** Michael S. Guzman, David Reed, Yoshiko Fujita, Yongqin Jiao, Dan M. Park

**Affiliations:** a Critical Materials Institute, Physical and Life Sciences Directorate, Lawrence Livermore National Laboratory, Livermore, California, USA; b Critical Materials Institute, Biological & Chemical Science & Engineering Department, Idaho National Laboratory, Idaho Falls, Idaho, USA; Indiana University, Bloomington

## Abstract

We report the complete genome sequence of Acidithiobacillus ferriphilus GT2, an acidophile isolated from gold mill tailings. The circular genome of GT2 contains 2,489 predicted protein-coding units and a single plasmid. Functional analysis indicates the metabolic potential to oxidize iron and reduced sulfur compounds and to fix N_2_ and CO_2_.

## ANNOUNCEMENT

Acidithiobacillus ferriphilus species include iron- and sulfur-oxidizing autotrophic bacteria isolated from metal-rich, acidic environments, such as acid mine drainage sites or hydrothermal pools (1). Due to their metabolic versatility and high tolerance to transition metals and salts, strains of *A. ferriphilus* have been proposed for industrial use in the bioleaching of sulfidic ores (1, 2). However, no complete and finished genome sequences of *A. ferriphilus* are currently available in public databases. We isolated, sequenced, and annotated the genome of *A. ferriphilus* GT2 to better understand its metabolic potential and the mechanistic underpinnings of its high metal and salt tolerance.

*A. ferriphilus* GT2 was isolated from gold mill tailings (Colorado, USA). A 1-g wet tailing sample was inoculated in 10 mL DSMZ medium 882 (https://www.dsmz.de/microorganisms/medium/pdf/DSMZ_Medium882.pdf). Enrichments were incubated at 30°C for 3 weeks and then diluted 1:100 into fresh media. GT2 was isolated on solid agar plates of 2:2 medium (3). Subsequently, GT2 was routinely cultivated in DSMZ medium 882, including for the harvesting of cells for DNA isolation. The FastDNA spin kit for soil (MP Biomedicals) was used to extract DNA according to the manufacturer’s recommendations. The size of genomic DNA was determined using gel electrophoresis (E-Gel SizeSelect II agarose gel; Invitrogen), which revealed a fluorescent band of >15 kb. The sample was then sheared using a g-TUBE device (Covaris, Inc.), and the average size of the sample was verified using the 2100 Bioanalyzer instrument (Agilent Technologies). Approximately 1 μg of genomic DNA was used as input for library preparation using the SMRTbell Express template prep kit 2.0 (Pacific Biosciences). During library preparation, the sample underwent DNA damage and end repair as well as barcode adapter ligation. Following library preparation, DNA fragments ranging from 6 kb to 10 kb were selected using the Sage Science BluePippin automated size-selection instrument. The final library concentration was measured using the Qubit double-stranded DNA (dsDNA) high-sensitivity (HS) assay kit (Thermo Fisher Scientific), and the average library size (7,668 bp) was determined using the Agilent 2100 Bioanalyzer instrument. The library was then subjected to circular consensus sequencing (CCS) using a 15-h movie time on the PacBio Sequel IIe instrument to generate HiFi reads. This resulted in 40,250 HiFi reads with a mean size of ∼6,756 bp. Preassembly read quality control (QC) was performed using the SMRT Link Run QC module. The CCS analysis workflow (SMRT Link software; Pacific Biosciences) was used to trim reads of adapters and compute consensus sequences from multiple passes around a circularized single DNA molecule (SMRTbell template). *De novo* assembly of the genome into 2 contigs (*N*_50_ contig length, 2,524,963 bp) was accomplished using the Improved Phase Assembler (IPA) via SMRT Link 10.1.0. IPA consists of several key processes, including overlapping (pancake), phasing (nighthawk), filtering overlaps (falcon), contig construction (falcon), and polishing (racon). Default parameters were used except where otherwise noted. The NCBI Prokaryotic Genome Annotation Pipeline (PGAP) was used for sequence annotation. Average nucleotide analysis (ANI) was computed in Anvi’o 7 (4, 5) using PyANI (6).

GT2 was identified as a strain of *A. ferriphilus* based on its 98.5% average nucleotide identity with the BLAST method (ANIb) and 100% 16S rRNA nucleotide identity to *A. ferriphilus* DSM 100412. Whole-genome sequencing resulted in two finished sequences, including a circular chromosome (2,524,963 bp; GC content, 57%) and a single plasmid (14,357 bp; GC content, 51%). The genome is 98.59% complete with a total of 2,489 coding DNA sequences (CDSs). Functional analysis indicated the metabolic potential to oxidize ferrous iron and reduced sulfur compounds as energy sources and to fix N_2_ and CO_2_ via the *nif* regulon and the Calvin-Benson-Bassham (CBB) cycle, respectively. Additionally, a variety of putative metal resistance genes were annotated, including those for arsenic, copper, mercury, cobalt, zinc, cadmium, and iron. The isolation and complete genome sequence of *A. ferriphilus* GT2 may provide a valuable resource for improving the utility of *A. ferriphilus* strains in biomining applications and increasing our understanding of microbial communities implicated in acid mine drainage.

### Data availability.

The PacBio genome sequence has been deposited in GenBank under the BioProject accession number PRJNA751914, the BioSample accession number SAMN20559762, the SRA accession number SRR15345381, and the GenBank accession numbers CP080536 and CP080537.
